# Primary Spinal Epidural Abscesses Not Associated With Pyogenic Infectious Spondylodiscitis: A New Pathogenetic Hypothesis

**DOI:** 10.3389/fsurg.2020.00020

**Published:** 2020-04-30

**Authors:** Lorenzo Magrassi, Marco Mussa, Andrea Montalbetti, Marta Colaneri, Angela di Matteo, Antonello Malfitano, Anna Maria Simoncelli, Maria Grazia Egitto, Claudio Bernucci, Enrico Brunetti

**Affiliations:** ^1^Neurosurgery, Department of Clinical, Surgical, Diagnostic and Pediatric Sciences, University of Pavia, IRCCS San Matteo Hospital Foundation, Pavia, Italy; ^2^Unit of Infectious and Tropical Diseases, Department of Clinical, Surgical, Diagnostic and Pediatric Sciences, IRCCS San Matteo Hospital Foundation, University of Pavia, Pavia, Italy; ^3^Neurosurgery Pope John XXIII Hospital, Bergamo, Italy; ^4^Unit of Infectious and Tropical Diseases, IRCCS Policlinico San Matteo Hospital Foundation, Pavia, Italy; ^5^Diagnostic Radiology, Interventional Radiology and Neuroradiology Unit, IRCCS Policlinico San Matteo Hospital Foundation, Pavia, Italy; ^6^Radiology Unit, Voghera Hospital, Voghera, Italy

**Keywords:** spinal epidural abscess, pyogenic infectious spondylodiscitis, epidural space, lymphatic vessels, spinal cord decompression, percutaneous treatment, laminectomy

## Abstract

**Introduction:** Spinal epidural abscess (SEA) incidence is rising. However, most series do not differentiate between SEAs associated with pyogenic infectious spondylodiscitis (PS) and SEAs limited to the epidural space.

**Methods:** We retrospectively reviewed the records and radiological images of all patients admitted to our institutions with a diagnosis of SEA not associated with PS between January 2013 and December 2018.

**Results:** We found three males and four females; five of the seven were intravenous drug users. All patients presented with pain: in six, it was associated with acute motor and sensory deficits, while one had only pain and paresthesias. *Staphylococcus aureus* was cultured from abscesses and/or from multiple blood cultures in four patients. Abscesses were localized to the cervical spine in one patient, thoracic in three, lumbar in one, and in two, the SEAs involved multiple segments. All patients but one underwent urgent open surgery. This patient had a multisegmental abscess and was successfully treated by percutaneous aspiration when pain became intractable. After abscess evacuation, the neurological deficits improved in all patients except one. The patients that were treated without spine instrumentation did not develop delayed kyphosis or instability at follow-up.

**Conclusion:** Patients with SEAs not associated with PS are likely to present with pain and motor deficits, appear to benefit from urgent abscess evacuation, and seem to be less dependent on spine instrumentation to avoid delayed spinal deformities compared to SEA associated with PS. Finally, the lack of initial involvement of bone and intervertebral disks may suggest that at least some of the SEAs without PS originate from infection of epidural lymphatic vessels that are not present inside those structures.

## Introduction

Spinal epidural abscess (SEA) is an infection of the spine that results in the accumulation of purulent fluid in the epidural space. Initial signs and symptoms are non-specific, and this often leads to a delay in the diagnosis (1). Symptoms usually begin with spinal pain and evolve with a variable temporal lag to root pain and weakness followed by paralysis (2). Overall, the incidence of SEAs in developed countries is estimated to be between 0.2 and 2 cases for every 10,000 hospital admissions (3), and recent studies indicate that the incidence of SEAs is rising possibly due to an increase in active intravenous drug users (IVDUs) (4). While the majority of the authors agree that early surgical treatment with spinal cord decompression and drainage of SEA is indicated (5, 6), the number of patients intentionally treated conservatively is increasing and it is reaching values as high 57% of all patients affected by SEA (7). Despite emergency SEA evacuation and spinal cord decompression being considered as the treatment of choice (8), multiple studies based on retrospective case series failed to demonstrate that evacuation of SEAs within 24 h from the neuroradiological diagnosis is better than delayed surgery (4, 9). These results suggest that it is still unclear what distinguishes patients who will benefit from early surgical intervention from those who are better managed conservatively (10, 11). Moreover, despite the growing interest in the identification of reliable predictive factors allowing optimal therapeutic decisions for SEA, most studies still lump together different classes of SEAs. This may represent a confounding factor since primary SEAs seem to require fewer surgeries and shorter courses of antibiotics than secondary ones (12). Primary or spontaneous SEAs arise in patients without previous history of trauma, surgery, or percutaneous treatments that violated the integrity of the spinal epidural space (12, 13); otherwise, they are considered secondary. Among primary SEA, a further distinction can be made between SEAs that are associated with pyogenic infectious spondylodiscitis (PS) and SEAs that, at least at the time of the diagnosis, are not associated with neuroradiological signs of infection of the vertebrae and/or the corresponding disks (14). Approximately 37% of patients affected by PS will develop an epidural abscess (15), and this rate is not affected by aging (16). Primary SEAs not associated with PS account for 13.79–35.4% of all SEAs (14, 17) and are usually considered as due to the hematogenous spread of circulating bacteria or fungi into the epidural space (12). Not unexpectedly, they are often found in an IVDU, and *Staphylococcus aureus*, a common skin contaminant, is the predominant pathogen isolated from primary SEA not associated with PS (14). *Staphylococcus aureus* is also the most common causative agent in all other class of SEA. However, agents other than *S. aureus* have been more often isolated in primary SEA compared to secondary SEA (12). *Staphylococcus aureus* isolated from SEA in IVDUs are more often multidrug resistant compared to other populations of patients (4). Moreover, recent anatomical work in experimental animals (18, 19) has confirmed with modern techniques the presence of lymphatic vessels in the epidural space associated with spinal roots and the dura as previously shown by indirect methods also in humans (20). The presence of lymphatic vessels along the roots suggests that at least some SEA without PS may result from the infection of the lymphatic more than the epidural venous plexus. Distinguishing between primary SEAs that are not associated with PS from those secondary to PS may thus be important for clinical reasons such as selecting a surgical strategy with or without spinal instrumentation, and the length of antibiotic treatment that in the case of PS is currently protracted for 6–8 weeks (21). Moreover, that distinction will promote further investigations on the possible involvement in primary SEAs of the still poorly known lymphatic vessel in the epidural space. In selected cases of SEA without PS, the absence of bone structural alterations may be an important indication for percutaneous aspiration of the abscess content in order to decompress the spinal cord and obtain materials for microbiological studies (22). Percutaneous approaches have already been described as an alternative to open surgery in selected patients (22–24). In order to increase our knowledge of primary SEA without PS, we reviewed our series in the last 5 years. We found that the majority of patients in our series had a history of intravenous drug use and that a more conservative approach with decompression of the spinal cord without spinal instrumentation through open or percutaneous approaches halted the neurological decay with marked improvement of the neurological deficits in 80% of the patients, and this was obtained without delayed kyphosis or instability of the affected segments.

## Materials and Methods

The study period was January 2013 through January 2018. Minimal length of follow-up was 1 year. All patients admitted at two tertiary-care hospitals of the densely populated Lombardia Region in Italy (Fondazione I.R.C.C.S. Policlinico San Matteo, Pavia and Ospedale Papa Giovanni XXIII, Bergamo) with a diagnosis of SEA without PS were reviewed. A revision of all available neuroradiological investigations was performed to ensure the absence of PS in the initial investigations (either CT and/or MRI) obtained before surgical treatment. Neurological examination findings are summarized according to the American Spinal Injury Association/International Spinal Cord Society (ASIA/ISCOS) impairment scale with grades A–E assigned based on review of the initial presentation documentation. Timing and approach of surgical intervention were determined on a case-by-case basis by the attending neurosurgeon in collaboration with an infectious disease specialist.

## Results

### Demographic and General Clinical Results

**Figure 1:**
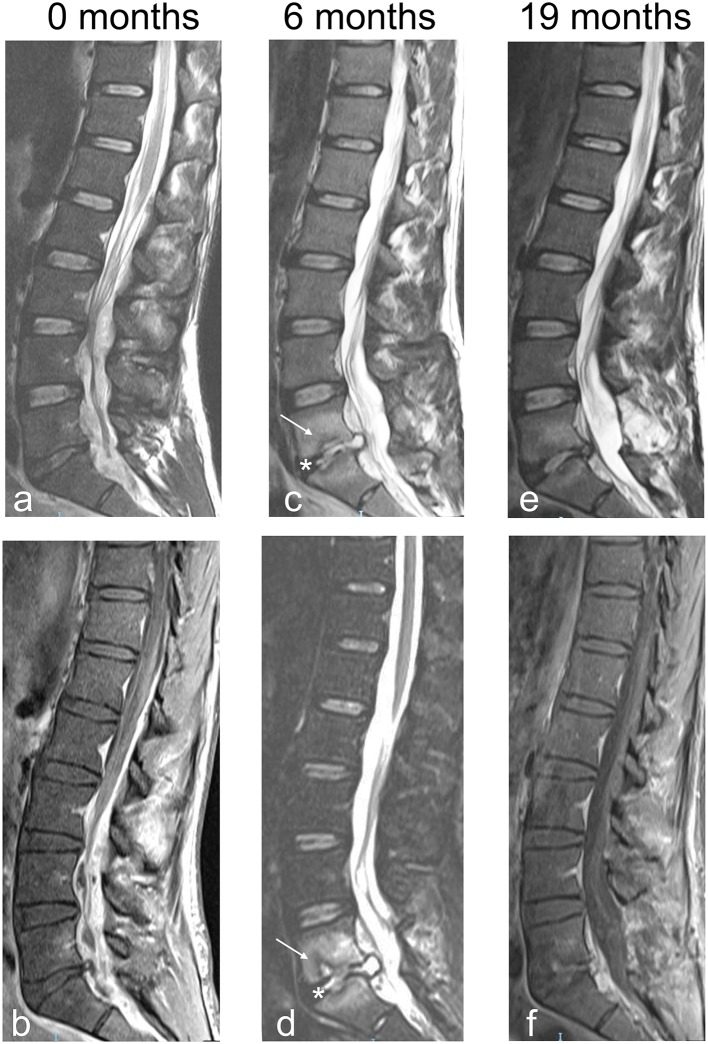
Sagittal MRI images of patient no. 2. **(a)** Sagittal MRI T2 weighted image obtained the day of surgery. There is a lack of obvious involvement of the bone despite a large SEA that circumferentially compresses the dural sac from L2 to S1. **(b)** Sagittal MRI T1 weighted with contrast. Same as in **(a)**. **(c)** Sagittal MRI T2 weighted image obtained 6 months after L5 laminectomy and SEA evacuation. Alterations compatible with PS involving the vertebral bodies of L4 and L5 (arrow) and the intervening disk (asterisk) are now visible. **(d)** Sagittal MRI T1 weighted with contrast. Same as in **(c)**. **(e)** Sagittal MRI T2 weighted image with contrast obtained 19 months after L5 laminectomy and SEA evacuation. Alterations related to PS have much regressed, without significant vertebral deformity. **(f)** Sagittal MRI T1 weighted with contrast. Same as in **(e)**.

A total of seven patients (three males and four females; four were treated in Pavia, and two in Bergamo) affected by primary SEA not associated with PS came to our attention in the 5 years of our study. Age in years ranged from 29.1 to 70.3 years (median age: 41.9 years); all patients except two were IVDUs and three had HIV infection. Demographics and information on their clinical status are presented in Table 1. All cases were evaluated by the attending neurosurgeon on an emergency basis due to severe pain or impending neurological deficits. All cases except one were treated surgically by open evacuation of the SEA within the first 12 h from the initial neuroradiological identification of SEA. The only case (case no. 3) that was treated by percutaneous aspiration 11 days after the initial diagnosis of an extensive posterior abscess was initially asymptomatic except for mild lumbosacral pain. Inability to stand and walk significantly increased thrombotic risk in these patients. In order to prevent thrombotic complications, we treated all patients with intermittent pneumatic compression devices during surgery and perioperatively, and we always started low molecular weight heparin as soon as possible after surgery (25). The neurological status of all patients, except for patient 5 affected by an anterior cervical SEA, improved at the first follow-up visit at 1 month, and their neurological status remained stable or further improved at the last follow-up visit available (minimum 1 year after surgery). All patients that were treated without spine instrumentation did not develop delayed kyphosis or instability of the affected segments at follow-up. Patient no. 2 (see Figures 1a,b) 6 months after surgery for SEA at the lumbar level had MRI evidence of PS without new neurological deficits (see Figures 1c,d) but only lumbar pain. He refused further surgery and was treated conservatively restarting the antibiotic therapy with complete resolution of the infection (see Figures 1e,f). We illustrate the detailed clinical history of two representative patients of our series.

**Table 1:** Patients characteristics.

**Pt**.	**Sex**	**Age (years)**	**IVDU**	**Diabetes type II**	**Viral inf**.	**Level**	**AISG**	**Type of surgery**
1	M	46.3	Yes	Yes	HIV, HBV, HCV	post Th5-7	C	P decompression
2	M	31.6	Yes	No	HCV	L2-S1	C	P decompression
3	M	70.3	No	Yes	no	post Th7-S2	C	P percutaneous aspiration
4	F	41.9	Yes	No	HIV	post Th4-7	C	P decompression
5	F	41.5	Yes	No	HIV, HCV	ant C6-Th1	B	A decompression + A instrumentation
6	F	29.1	Yes	No	HCV	ant Th5-9 post Th9-L2	B	P decompression
7	F	65.6	No	No	no	post Th2-7	C	P decompression

*Study population characteristics with primary SEA not associated with PS. AISG, ASIA/ISNCSCI impairment scale grade; Pt, patient; C, cervical; Th, thoracic; L, lumbar; S, sacral; P, posterior; A, anterior. One patient (no. 6) had SEA extending from thoracic to lumbar spine, and the purulent collection becomes confluent around the dura at Th9*.

### Illustrative Cases

**Figure 2:**
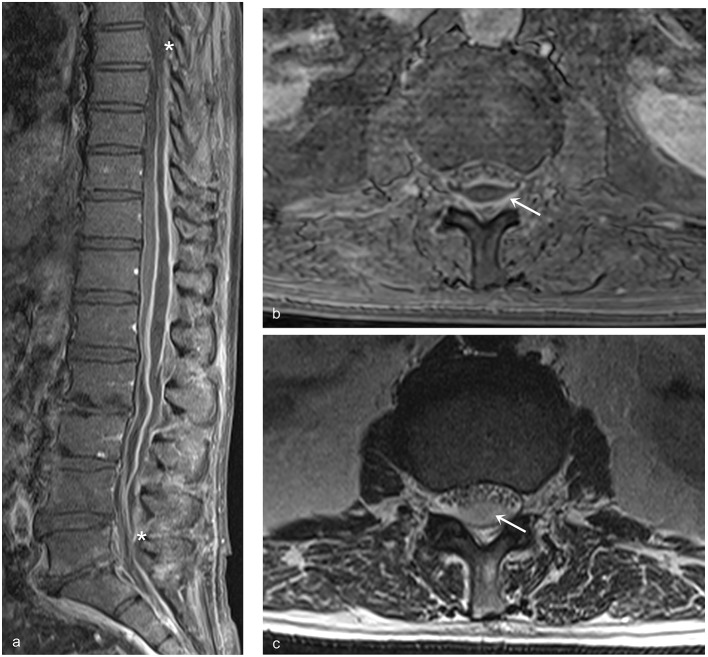
Sagittal and axial MRI images of patient no. 3. **(a)** The extensive posterior SEA extending from Th7 to L5 is delimited by an enhancing pseudo-capsule. The asterisks indicate the cranial and caudal limits of the lesion compressing anteriorly both the spinal cord and the cauda equina. **(b)** Axial MRI image T1 weighted with contrast. The arrows indicate the enhancing capsule of the SEA; psoas and paravertebral muscles appear normal. **(c)** Axial MRI image T2 weighted. The arrow indicates the purulent content of the SEA; no edema is visible in psoas and paravertebral muscles.

**Figure 3:**
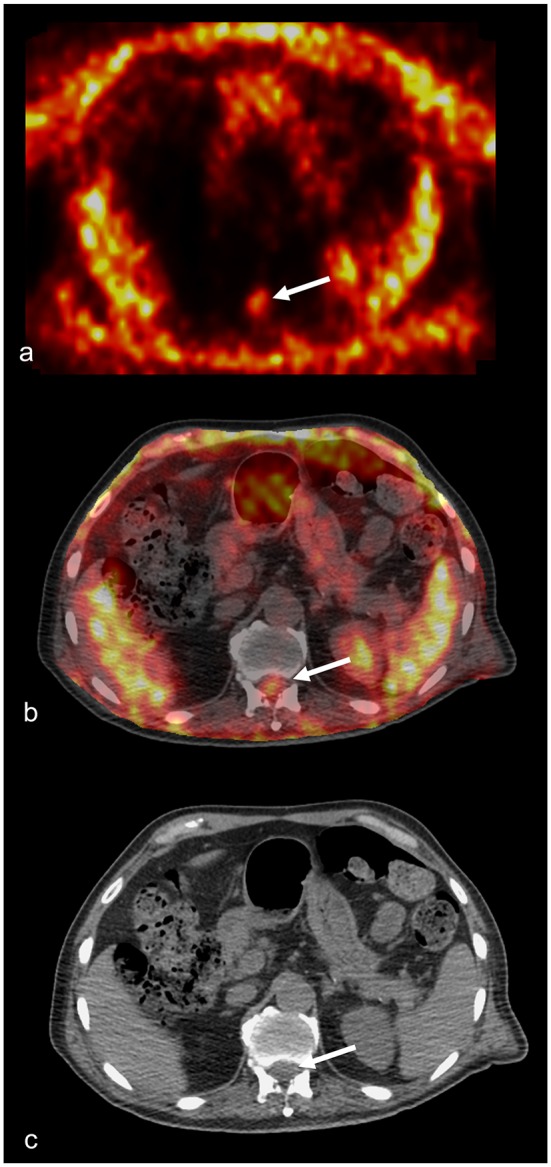
^18^FDG-PET and CT scan of patient no. 3. **(a)** ^18^FDG-PET axial section taken at the level of L4. The arrow points to the accumulation of the tracer corresponding to the SEA. **(b)** ^18^FDG-PET axial section fused to the corresponding CT scan image. The arrow point to the accumulation of the tracer corresponding to the SEA contained in the lumbar canal. There is minimal or no accumulation of the tracer into the vertebra or the paraspinal muscles. **(c)** Axial CT scan image used for the fusion shown in **(b)**. The arrow points to the accumulation of the tracer.

Case no. 3. A 70.3-year-old man with a history of hypertension, acute pancreatitis, and chronic kidney failure associated with complete resection of the bladder with bilateral ureterostomy for extensive local cancer. One month after surgery, he presented fever and low back pain that radiated bilaterally to the lower limbs. At the neurological evaluation, hypoesthesia on the outer face of proximal left leg and bilateral lower limbs hyporeflexia were found. A thoracolumbar contrast enhanced MRI of the spine showed an extensive epidural collection with compression of the spinal cord and the cauda equina extended from Th7 to S2 without signs of PS (see Figures 2a–c). An 18F-deoxyglucose PET scan confirmed lack of bone involvement with accumulation of the tracer limited to the epidural space (see Figure 3). Repeated blood and urine cultures resulted positive for *Staphylococcus warneri* and *S. aureus*, respectively. Due to the poor general conditions and the relatively mild neurological symptoms, combination therapy with piperacillin-tazobactam, trimethoprim-sulfametoxazole, ciprofloxacin, and cefazolin was begun and continued for 60 days. One week after starting the antibiotics, the pain exacerbated and became continuous despite the fact that the patient was continuously resting in bed. Pain resolved after percutaneous drainage of the abscess. Cultures were performed and the aspirated pus resulted negative.

Case no. 4. A 41.9-year-old active IVDU and HIV positive woman was admitted to our hospital after 3 days of progressively worsening low back and abdominal pain associated with paresthesias and tactile hypoesthesia of the lower limbs and inability to stand. On initial examination, she was mildly febrile, vital signs were stable, physical examination of the chest was normal, abdomen was distended, palpation revealed a diffuse tenderness, the bladder was distended, and catheterization demonstrated acute urinary retention (approximately 1 L of urine). Neurological examination showed mild nuchal rigidity, complete paralysis of the lower left limb, and paresis of the right lower limb (MRC scale 2); patellar reflex was bilaterally brisk associated on the left side with a few beats of unsustained clonus, the Achilles reflex was bilaterally normal, a positive Babinski sign was elicitable on both sides, and a sensory level of T10 was present.

**Figure 4:**
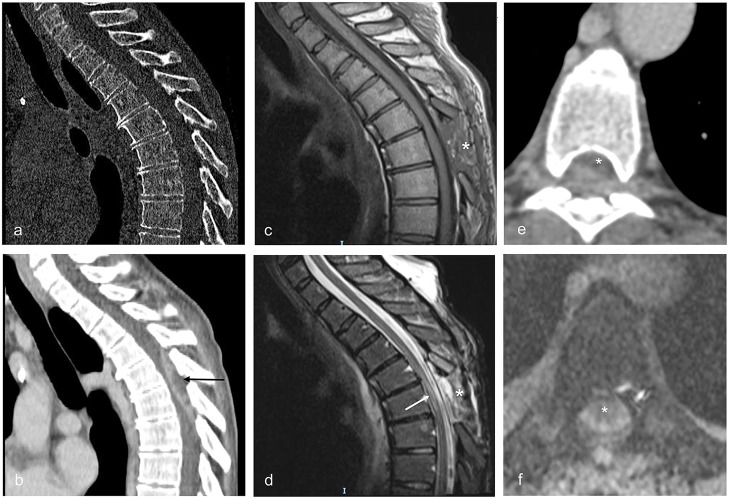
Sagittal and axial CT and MRI images illustrating patient no. 4. **(a,b)** Sagittal CT images obtained immediately before surgery showing the lack of bone alterations suggestive for PS and the posterior SEA extending from Th4 to Th7 (black arrow). **(c)** Sagittal MRI image T1 weighted with contrast obtained 3 days after surgery (enlarged Th5 laminectomy). The asterisk indicates the site of the laminectomy. **(d)** Sagittal MRI image T2 weighted obtained 3 days after surgery. The alterations of the spinal cord due to compression by the abscess are visible as irregular signal hyperintensities inside the cord (white arrow). The asterisk indicates the site of the laminectomy. **(e)** Axial contrast enhanced CT scan obtained immediately before surgery, showing the spinal cord (asterisk) compressed anteriorly by the abscess at Th5. **(f)** Axial MRI image T2 gradient echo obtained 3 days after surgery (enlarged Th5 laminectomy); compression of the spinal cord (asterisk) is relieved.

Laboratory findings revealed leukocytosis (15,000 WBC/mm^3^) with neutrophilia and an elevation of C-reactive protein concentration (16.45 mg/dl). Blood and urine cultures were obtained. Abdominal X-ray showed signs of coprostasis. HIV infection had been diagnosed 7 years before and blood sample taken on admission revealed the following: CD4 cells 255/mm^3^, HIV-RNA undetectable, and CD4/CD8 ratio 0.9. The patient was transferred to the Infectious Disease ward for further treatment. A lumbar puncture was performed and revealed the following: clear cerebrospinal fluid (CSF), mild pleocytosis (150 cell/mm^3^), glycorrhachia was 28 mg/dL with a glycemia of 92 mg/dl, and protein concentration was normal. Due to rapid neurological deterioration, an urgent contrast enhanced whole spine CT scan was obtained (see Figures 4a–d). The exam showed an epidural mass 6 cm long and 1 cm thick in the posterior half of the spinal canal extending between Th4 and Th7 with obvious compression of the spinal cord. An immediate surgical decompression of the neural elements and drainage of the abscess were performed. Afterward, an empirical antibiotic and antiviral therapy with oxacillin (2 g q.6h), imipenem (1gr q.8h), acyclovir (0.8 gr q6h), and steroids were started. Gram stains performed on the abscess drainage liquid revealed Gram-positive cocci, and a methicillin-sensitive *S. aureus* was later isolated both from the abscess and from multiple blood cultures. When the cultures and antibiogram results became available, imipenem and acyclovir were stopped and the therapy was de-escalated to oxacillin alone. After spinal cord decompression, the patient showed immediate signs of neurological improvement, with resolution of the paralysis and improvement in the severe hypoesthesia. A follow-up MRI, obtained 3 days after surgery, demonstrated a mild spinal postcompressive damage and no evidence of PS (see Figures 4e,f). She was transferred to a rehabilitation center where she continued the antibiotic therapy for 6 weeks. Her motor and sensory deficits progressively improved: at a follow-up visit 6 months later, she was able to walk autonomously with normal sphincter control.

## Discussion

Despite advances in medical and surgical management of SEAs, they are still associated with substantial morbidity and mortality with 8% stable paraparesis and 7% of death even in recent series (26). Surgical decompression with or without the use of intraoperative ultrasound (27) is considered important in SEA management, but a recent meta-analysis showed that the number of patients primary treated by medical therapy has increased to ~40% and the same authors did not find any statistically significant difference between surgical and non-surgical management of SEAs in terms of patient outcome (5). Others concluded that surgery with adjuvant antibiotic therapy is more likely to result in neurological recovery in patients with neurologically symptomatic SEAs (28). These contradictory results indicate that further research and a more detailed classification of the various types of SEA are needed to clarify the outcome predictors and the indications of surgical vs. non-surgical therapy for SEA (28). An important predictor of the outcome of SEA is whether or not they are associated with PS. Primary SEAs not associated with PS are rare compared to other pyogenic infections of the vertebrae and the spinal cord (1, 29) and represent ~20% of all SEAs. Unfortunately, most meta-analyses and the largest published case series do not distinguish between primary SEA associated and not associated with PS, and little is known of their natural history. In our series, we confirmed by CT, MRI, and PET scan that primary SEA may sometimes develop before infection of the adjacent vertebrae and disks. At least at the thoracolumbar level, the presence of posterior paraspinal muscle edema is more sensitive and specific for SEA associated with PS compared to bone marrow edema, psoas edema, and intervertebral disk signal abnormality in isolation (30). Paraspinal muscle edema was not present in patients in our series even in those affected by thoracolumbar SEA. This is another indication that SEA may sometimes occur without initial involvement of the adjacent bone and ligaments. The route of infection for primary SEAs not associated with PS is usually considered hematogenous (3). SEAs are frequently associated with moderate to marked dilatation of the spinal epidural venous plexus (31). However, despite the lack of valves in the radicular veins separating the epidural venous plexus from the intradural venous compartments and the physiological presence of reflux flow through the spinal radicular veins under conditions that increase the venous pressure in the epidural plexus (32), none of our patients had any evidence of spreading of the infection from the epidural to the intradural compartment, and in general, intradural spinal abscesses are very rare (33). Interestingly, while the epidural venous plexus is also extensively connected with the veins in the vertebral bone, epidural lymphatics follow the nerve roots and do not enter the vertebrae or the disk space that are devoid of lymphatics (34–36). This supports the idea that at least some of SEAs without PS originate within the lymphatic vessels associated with the nerve roots (20) bypassing the bone and epidural venous plexus that may become involved only secondarily by contiguity. Spreading of infection by contiguity was exemplified by our patient number 4 who, following poor compliance to antibiotic therapy, developed PS in one of the involved segments a few months after surgical evacuation of the abscess. Primary SEAs have been associated with a wider range of pathogens than secondary SEAs, and they are also often associated with a wider range of concurrent diseases (12). We analyzed our case series of SEAs not associated with pyogenic infections of the vertebrae and intervertebral disks excluding SEAs secondary to surgical or percutaneous treatments that violated the epidural space. In our series of primary SEAs without concomitant PS, 71.42% of the patients had a history of active intravenous drug use, and three of these (60%) were also people living with HIV (PLHIV). Intravenous drug use is already considered a risk factor for primary SEA (4); moreover, intravenous drug use in PLHIV is associated with altered monocyte function and a dysregulated innate cytokine response potentially leading to an increased risk of infection (37, 38). Our series suggests that the association between primary SEA not associated with PS and IVDU may be even stronger than for all primary SEAs considered together. One of the non-IVDU patients in our series had multisegmental SEA that extended from Th7 to S2; he was not severely immunocompromised, but he had bilateral uretrostomies and chronic renal failure associated with recurrent urinary infections due to multiple *Staphylococcus* species. Interestingly, patients with recurrent *S. aureus* bacteriuria and bacteremia have a higher rate of spinal infections than patients with only *S. aureus* bacteremia (39). The association of recurrent *S. aureus* urinary infections with spinal infection may result from retrograde dissemination through the pelvic venous and lymphatic vessels that are connected to the intraspinal plexus. The same patient affected by a multisegmental SEA had only moderate pain localized to the spine and mild sensory deficits without motor impairment and was treated by antibiotics and percutaneous aspiration of the SEA with good results. Non-operative management of SEA has been successfully adopted in patients with a stable neurological condition (24, 40) or with SEA affecting multiple segments of the column (41). Percutaneous aspiration of the abscess for decompression of the spinal cord and culture is safe and effective in selected patients (23, 24). Moreover, panspinal infections are often associated with poor general conditions increasing the risk of open surgery and may be treated medically (42). All our patients, except the patient with the cervical SEA that remained paraplegic, had a significant improvement in their neurological conditions after abscess evacuation. Our results are in line with previous data and suggest that even patients with severe neurological deficits due to primary SEA without PS, when appropriately treated, have better chances of recovery after completing an inpatient rehabilitation program compared to patients affected by traumatic spinal cord injury (43) or secondary SEAs (12) or SEAs associated with PS (17).

## Limitations

Limitations of the present study include its retrospective character and the small number of patients considered. These limitations are partially outweighed by the multicentric nature of the study and its specific focus on patients with primary SEAs without concomitant vertebral and/or ligamentous involvement. This group of patients notoriously represents only a small minority of all SEAs (3, 14), and in the large majority of the studies, these patients were not recognized as separate despite their peculiar characteristics.

## Conclusion

Differentiation between primary SEA without association to PS and SEA originating from PS is necessary to improve our knowledge of the early stages of the infection that leads to such a different tissue involvement, and it is important both for prognostic and therapeutic decisions.

## Data Availability Statement

All datasets generated for this study are included in the article/Supplementary Material.

## Ethics Statement

All procedures performed in studies involving human participants were in accordance with the ethical standards of the institutional and/or national research committee and with the 1964 Helsinki declaration and its later amendments or comparable ethical standards. For this type of study, formal consent is not required.

## Author Contributions

LM and EB designed the study. All authors collected, assembled and analyzed the data. Project planning was performed by LM, EB, and CB. LM wrote the manuscript. All authors read, edited and approved the manuscript.

## Conflict of Interest

The authors declare that the research was conducted in the absence of any commercial or financial relationships that could be construed as a potential conflict of interest.
